# Supplementary material for: Ferrochelatase is a therapeutic target for ocular neovascularization
Source: EMBO Mol Med. 2017 Apr 4;9(6):786–801. doi: 10.15252/emmm.201606561 (PMC5452042; doi:10.15252/emmm.201606561)
Supplement: Supplementary file 1 — Appendix [file EMMM-9-786-s001.pdf]

Appendix

Ferrochelatase is a therapeutic target for ocular neovascularization

Halesha D. Basavarajappa[1,2], Rania S. Sulaiman[1,3,4], Xiaoping Qi[1], Trupti Shetty[1,3], Sardar Sheik Pran Babu[1], Kamakshi L. Sishtla[1], Bit Lee[5], Judith Quigley[1], Sameerah Alkhairy[1], Christian M. Briggs[1], Kamna Gupta[1], Buyun Tang[2], Mehdi Shadmand[1], Maria B. Grant[1,3], Michael E. Boulton[1], Seung-Yong Seo[5] & Timothy W. Corson[1,2,3]*

[1]Eugene and Marilyn Glick Eye Institute and Department of Ophthalmology, Indiana University School of Medicine, Indianapolis, IN 46202, USA.
[2]Department of Biochemistry and Molecular Biology, Indiana University School of Medicine, Indianapolis, IN 46202, USA.
[3]Department of Pharmacology and Toxicology, Indiana University School of Medicine, Indianapolis, IN 46202, USA.
[4]Department of Biochemistry, Faculty of Pharmacy, Cairo University, Cairo, Egypt.
[5]College of Pharmacy, Gachon University, Incheon 406-840, South Korea.

*To whom correspondence should be addressed: tcorson@iu.edu

Table of Contents

Appendix Supplementary Figures 2
Appendix Figure S1. Peptide mass fingerprinting analysis 2
Appendix Figure S2.
Effect of *FECH* knockdown on mRNA expression in HRECs 3

Accession	Description	Score	Coverage	# Unique Peptides	# Peptides	# PSMs*
F1S1X4	Ferrochelatase (Fragment) OS=Sus scrofa GN=FECH PE=3 SV=2 - [F1S1X4_PIG]	62.5	34.4	9	9	59
P00761	Trypsin OS=Sus scrofa PE=1 SV=1 - [TRYP_PIG]	58.0	8.7	1	1	80
F1RUV5	Uncharacterized protein (Fragment) OS=Sus scrofa GN=PC PE=4 SV=2 - [F1RUV5_PIG]	27.0	5.7	3	3	25
I3LVD5	Actin, cytoplasmic 1 OS=Sus scrofa GN=ACTB PE=2 SV=1 - [I3LVD5_PIG]	26.9	14.7	3	3	16
I3LNT6	Uncharacterized protein OS=Sus scrofa GN=KRT77 PE=3 SV=1 - [I3LNT6_PIG]	21.7	3.8	2	2	24
I3LLY8	Uncharacterized protein OS=Sus scrofa GN=KRT79 PE=3 SV=1 - [I3LLY8_PIG]	18.8	4.5	1	2	7
F1SGI7	Uncharacterized protein (Fragment) OS=Sus scrofa GN=LOC100525745 PE=3 SV=2 - [F1SGI7_PIG]	18.2	3.9	1	2	5
F1SHC1	Uncharacterized protein OS=Sus scrofa GN=LOC100127131 PE=3 SV=1 - [F1SHC1_PIG]	12.8	5.6	2	2	5

* PSMs → Peptide Spectrum Match

Appendix Figure S1. Peptide mass fingerprinting analysis of proteins pulled down with a cremastranone affinity reagent from a porcine brain lysate.

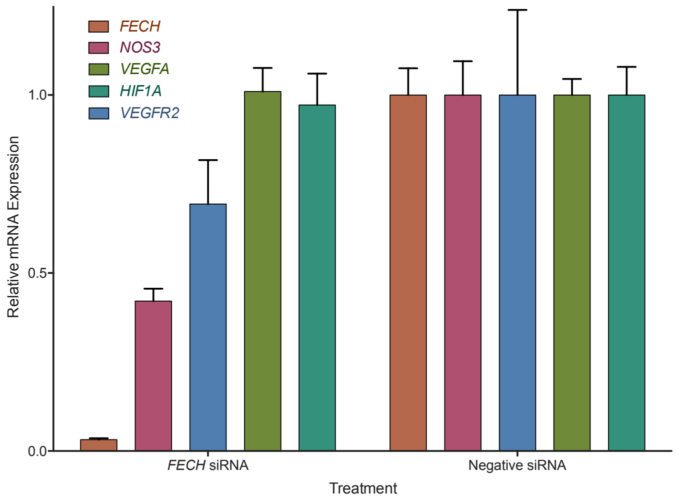

Appendix Figure S2. Effect of *FECH* knockdown on mRNA expression in HRECs.

qRT-PCR analysis shows no decrease in *VEGFA* or *HIF1A* expression while *NOS3* (eNOS) and *VEGFR2* expression are decreased after *FECH* knockdown.

Data information: Graph shows mean ± SD with n = 3 technical replicates for each group. Representative result from three independent experiments.
