# Supplementary material for: Ferrochelatase is a therapeutic target for ocular neovascularization
Source: EMBO Mol Med. 2017 Apr 4;9(6):786–801. doi: 10.15252/emmm.201606561 (PMC5452042; doi:10.15252/emmm.201606561)
Supplement: Supplementary file 2 — Expanded View Figures PDF [file EMMM-9-786-s002.pdf]

Expanded View Figures

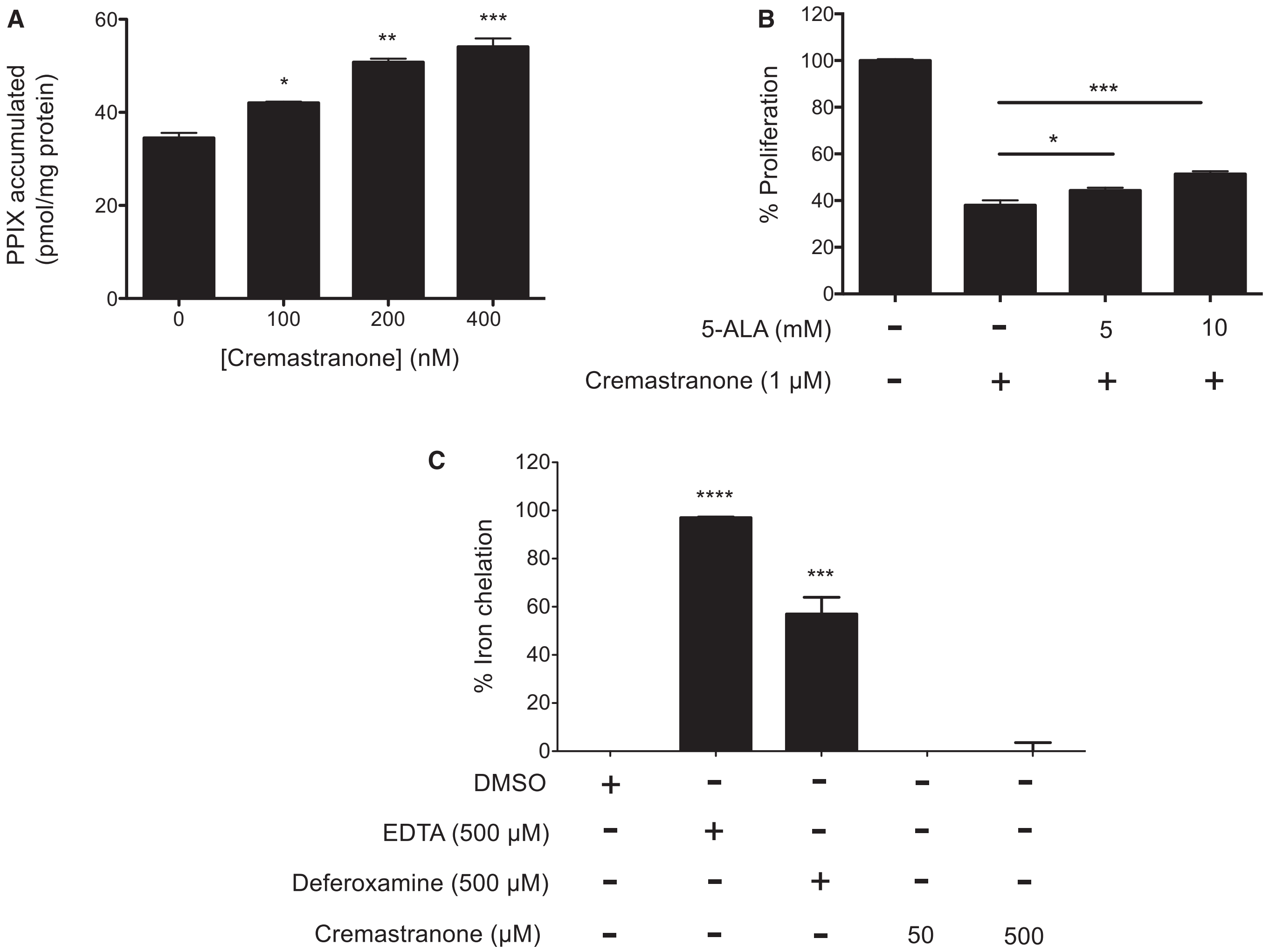

Figure EV1. Validation of cremastranone's inhibition of FECH.

A 5-Aminolevulinic acid (5-ALA)-induced protoporphyrin IX (PPIX) buildup in HRECs after cremastranone treatment. *P = 0.0201; **P = 0.0012; ***P = 0.0006 relative to no cremastranone control, ANOVA with Dunnett's *post hoc* tests (n = 3 per group).

B Partial rescue of cremastranone's inhibition of HREC proliferation with 5-ALA, an inducer of heme biosynthesis. HRECs treated with DMSO only are shown as 100% proliferation control. *P = 0.046; ***P = 0.0006, ANOVA with Tukey's *post hoc* tests (n = 3 per group).

C Cremastranone does not bind iron as determined in an iron chelation assay; EDTA and deferoxamine are positive controls. ****P = 0.0001; ***P = 0.0003 relative to DMSO control, ANOVA with Dunnett's *post hoc* tests (n = 2 per group).

Data information: Representative figures from at least three independent experiments. Graphs show mean ± SEM.

© 2017 The Authors

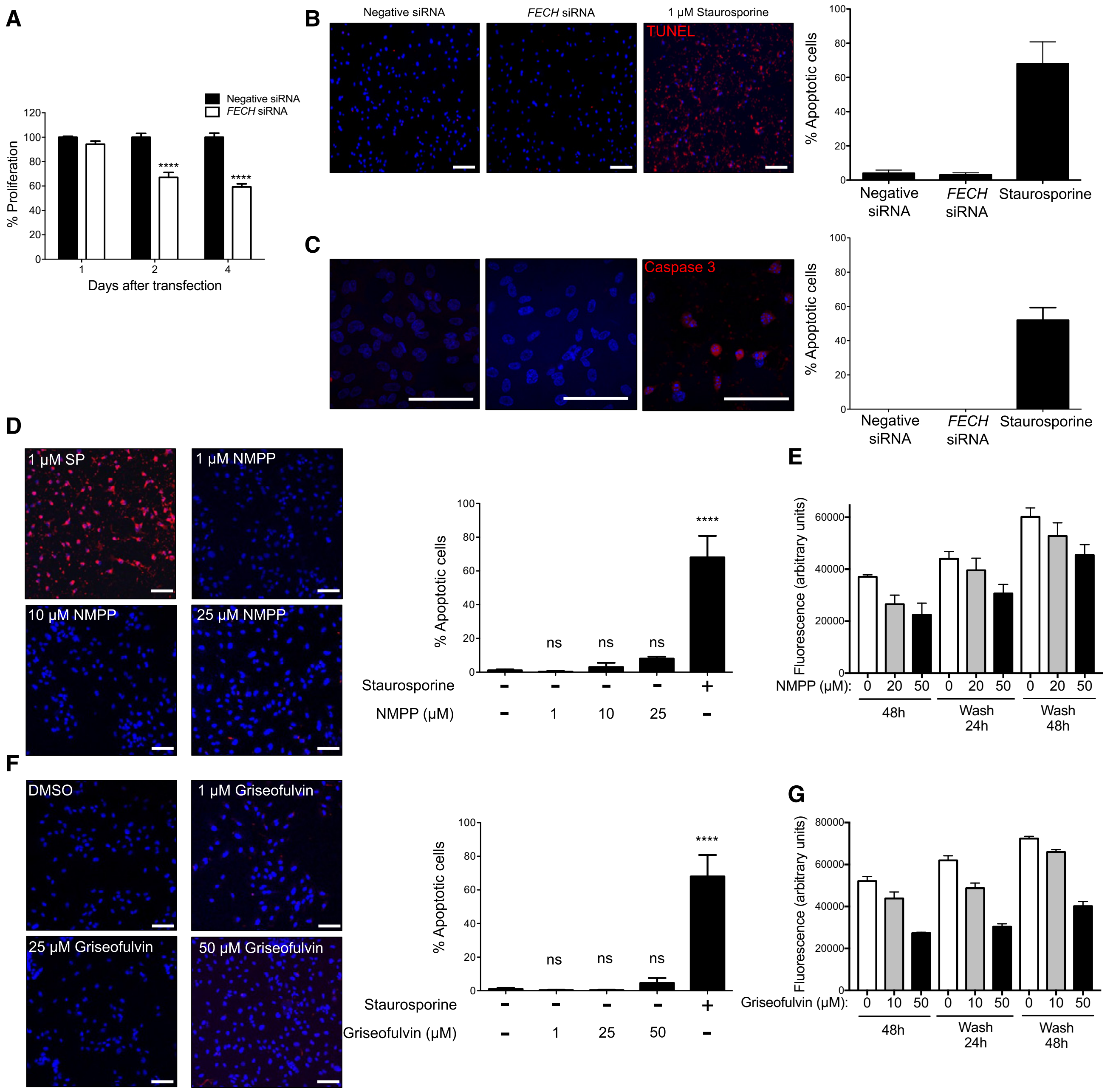

Figure EV2. Effects of FECH inhibition on HRECs.

A Time course of the effect of *FECH* siRNA on proliferation of HRECs. The % proliferation calculated are with respect to proliferation with negative control siRNA. ****P = 0.0001 versus negative siRNA at same time point, two-way ANOVA with Bonferroni's *post hoc* tests.

B *FECH* knockdown does not induce apoptosis, as assessed by TUNEL (red). Staurosporine (1 µM) is a positive control.

C *FECH* knockdown does not induce apoptosis, as assessed by activated caspase-3 immunostaining (red). Staurosporine (1 µM) is a positive control.

D Apoptosis of HRECs after treatment with different doses of NMPP as assessed by TUNEL assay. ns, not significant; ****P = 0.0001 as compared with no treatment group, ANOVA with Tukey's *post hoc* tests. Staurosporine (SP) is positive control.

E Washout of NMPP reverses antiproliferative effects. HRECs were treated for 48 h with the indicated concentrations of NMPP; then, drug was removed and proliferation assessed 24 and 48 h later by AlamarBlue.

F Apoptosis of HRECs after treatment with different doses of griseofulvin as assessed by TUNEL assay. ns, not significant; ****P = 0.0001 as compared with no treatment group, ANOVA with Tukey's *post hoc* tests.

G Washout of griseofulvin reverses antiproliferative effects. HRECs were treated for 48 h with the indicated concentrations of griseofulvin; then, drug was removed and proliferation assessed 24 and 48 h later by AlamarBlue.

Data information: Representative figures from at least three independent experiments. Graphs show mean ± SEM, n = 3. Scale bars = 1 mm.

 © 2017 The Authors

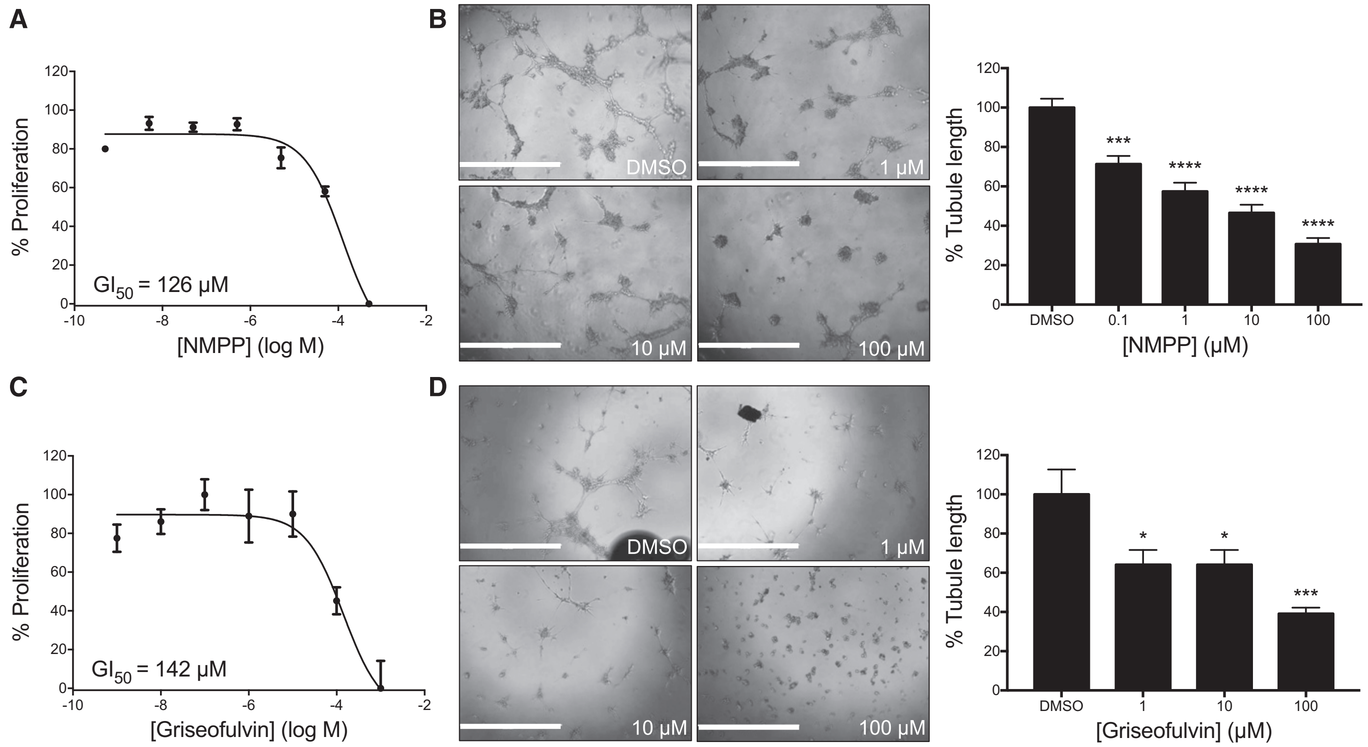

Figure EV3. Effects of FECH inhibition on Rf/6a choroidal endothelial cells.

A The effect of NMPP, a specific inhibitor of FECH activity, on *in vitro* proliferation was measured using an AlamarBlue assay (n = 3 per dose).

B Ability of NMPP-treated Rf/6a cells to form tubular structures in Matrigel was monitored and analyzed using ImageJ. ***P = 0.0002; ****P = 0.0001 compared to DMSO-treated sample, ANOVA with Dunnett's *post hoc* tests (n = 6 per group).

C The effect of griseofulvin on *in vitro* proliferation was measured using an AlamarBlue assay (n = 3 per dose).

D Ability of griseofulvin-treated Rf/6a cells to form tubular structures in Matrigel was monitored and analyzed using ImageJ. *P = 0.018; ***P = 0.0002 compared to DMSO-treated sample, ANOVA with Dunnett's *post hoc* tests (n = 6 per group).

Data information: Representative figures from at least three independent experiments. Graphs show mean ± SEM. Scale bars = 1 mm.

© 2017 The Authors

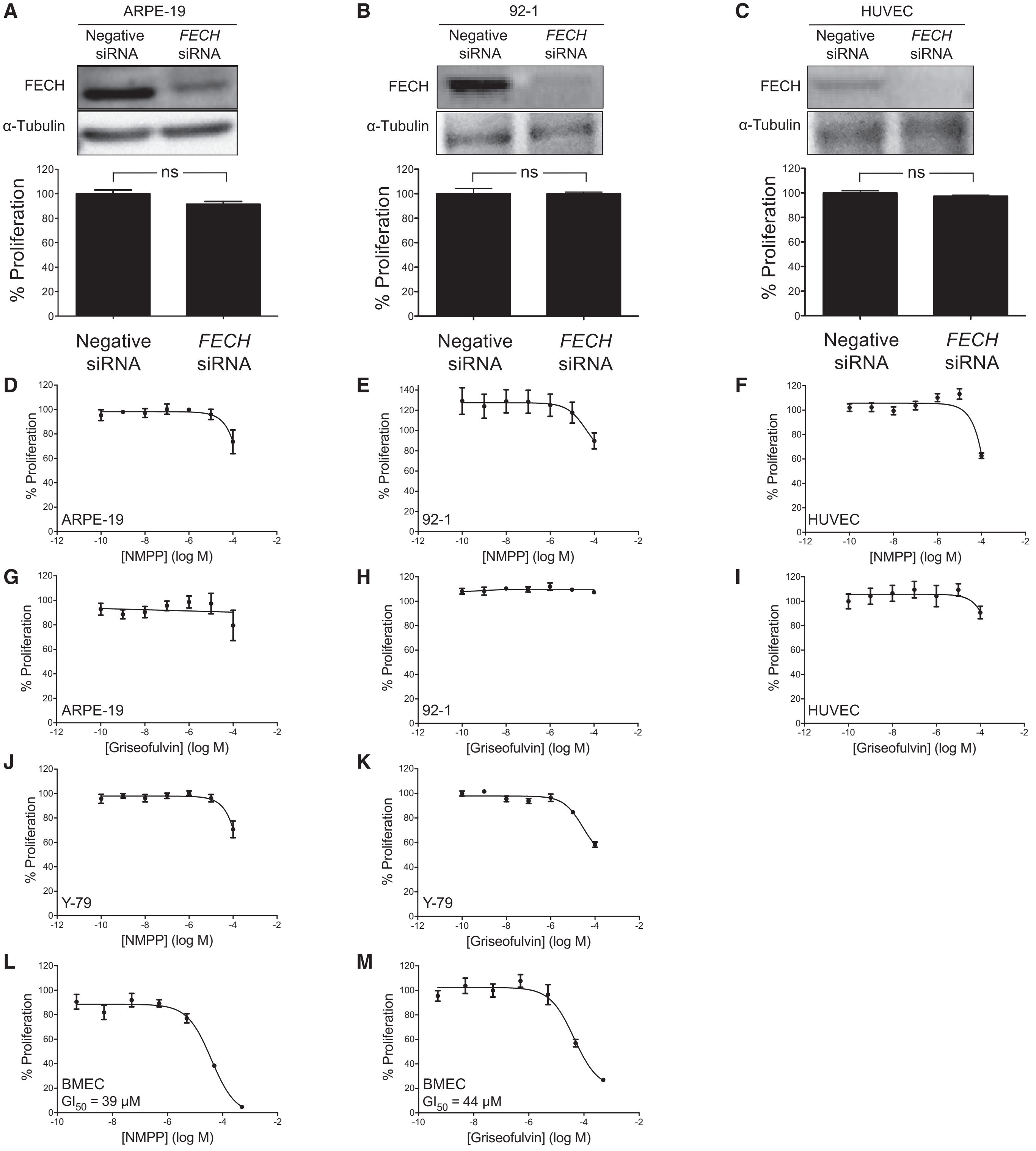

Figure EV4.

 © 2017 The Authors

Figure EV4. *FECH* knockdown or inhibition has no significant effects on proliferation of other ocular or macrovascular cell types.

- A Effect of *FECH* knockdown on proliferation of ARPE-19 retinal pigment epithelial cells. ns, non-significant, $P > 0.05$, two-tailed unpaired Student's *t*-test.
- B Effect of *FECH* knockdown on proliferation of 92-1 uveal melanoma cells. ns, non-significant, $P > 0.05$, two-tailed unpaired Student's *t*-test.
- C Effect of *FECH* knockdown on proliferation of human umbilical vein endothelial cells (HUVECs). ns, non-significant, $P > 0.05$, two-tailed unpaired Student's *t*-test.
- D Effect of NMPP on proliferation of ARPE-19 cells.
- E Effect of NMPP on proliferation of 92-1 cells.
- F Effect of NMPP on proliferation of HUVECs.
- G Effect of griseofulvin on proliferation of ARPE-19 cells.
- H Effect of griseofulvin on proliferation of 92-1 cells.
- I Effect of griseofulvin on proliferation of HUVECs.
- J Effect of NMPP on proliferation of Y-79 retinoblastoma cells.
- K Effect of griseofulvin on proliferation of Y-79 cells.
- L Effect of NMPP on proliferation of human brain microvascular endothelial cells (BMECs).
- M Effect of griseofulvin on proliferation of BMECs.

Data information: Graphs show mean ± SEM, $n = 3$. Representative figures from three experiments are shown in (A, B, and C).
Source data are available online for this figure.

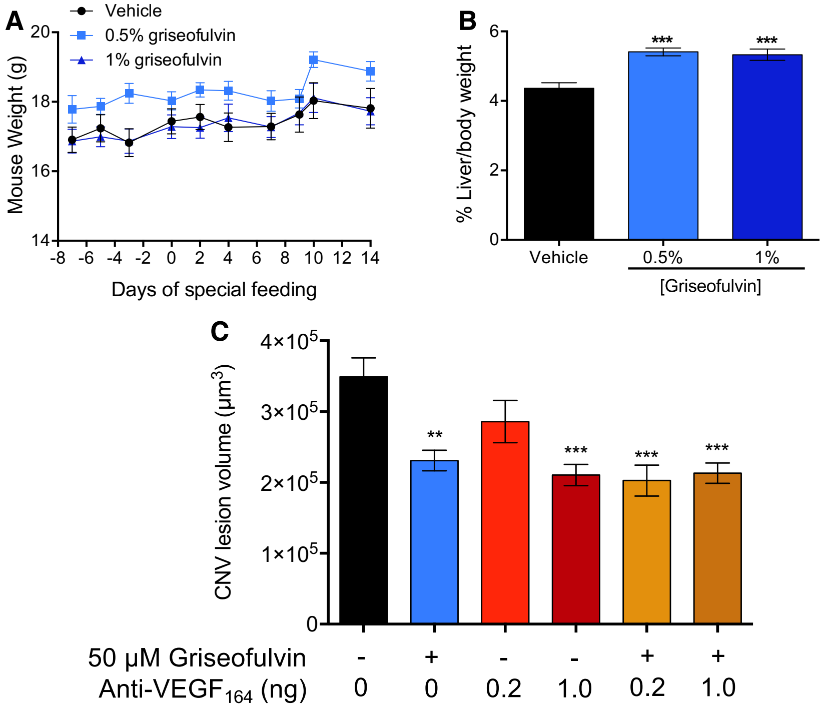

Figure EV5. Oral griseofulvin's systemic effects and combination with anti-VEGF therapy.

- A Oral griseofulvin treatment did not significantly change mouse weights during the experimental time course. $P > 0.05$, two-way repeated measures ANOVA with Tukey's *post hoc* tests ($n = 10$ mice per group).
- B Griseofulvin increased liver weights as expected with these treatments, confirming drug intake and systemic metabolism. $^{***}P = 0.0001$ and 0.002 versus vehicle, ANOVA with Dunnett's *post hoc* tests ($n = 6$ mice per group).
- C Intravitreal griseofulvin in combination with anti-VEGF$_{164}$ therapy in L-CNV. Treatment with indicated single agents and combinations. $^{**}P = 0.0011$ versus vehicle; $^{***}P = 0.0002$, < 0.0001, 0.0001, versus vehicle, left to right, respectively, ANOVA with Tukey's *post hoc* tests. All other comparisons were non-significant ($n = 6$–10 mice per group).

Data information: Graphs show mean ± SEM.
