# Supplementary figures and images for: Ferrochelatase is a therapeutic target for ocular neovascularization
Source: EMBO Mol Med. 2017 Apr 4;9(6):786–801. doi: 10.15252/emmm.201606561 (PMC5452042; doi:10.15252/emmm.201606561)

Fig. EV 4A

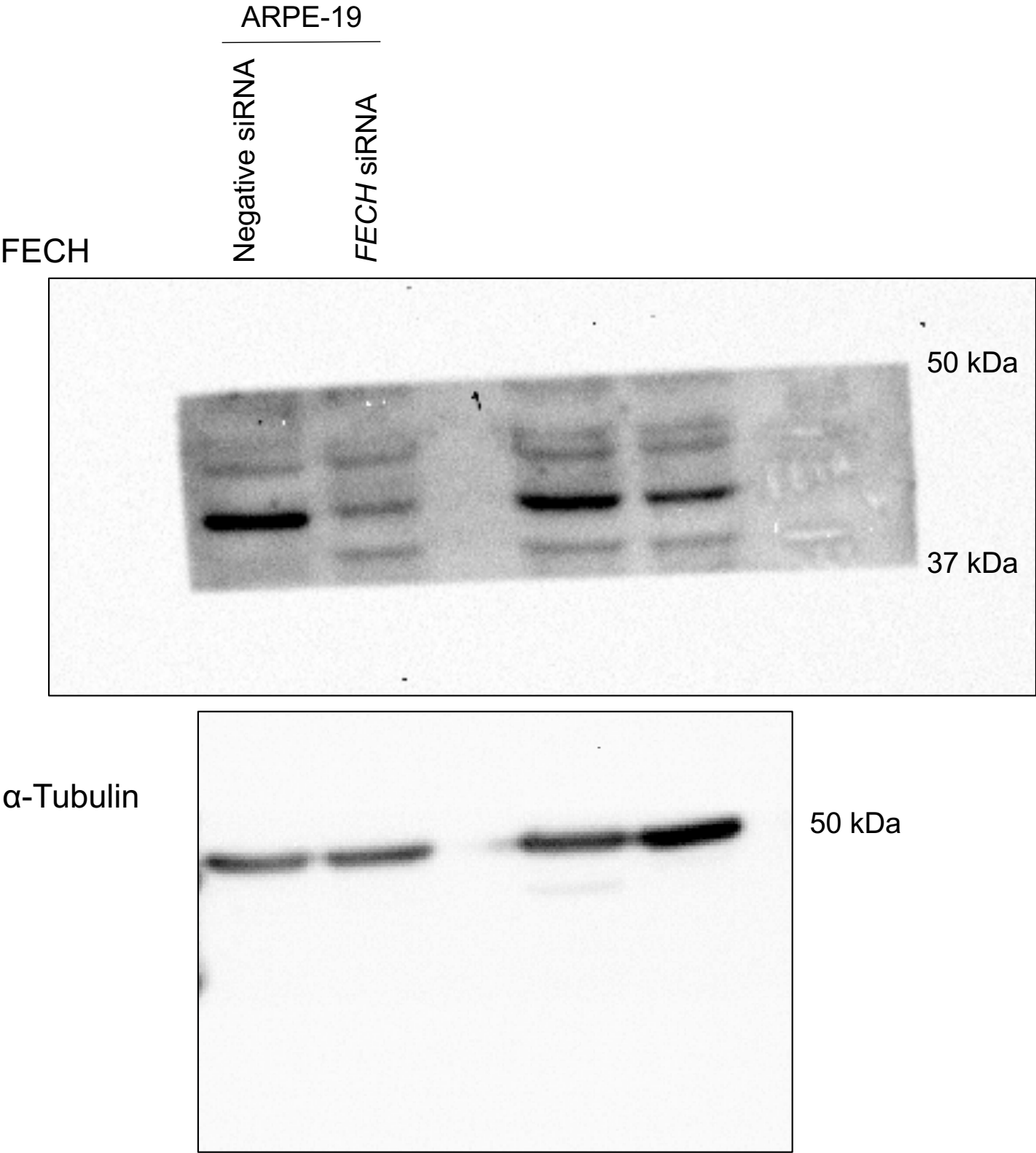

Fig. EV 4B,C

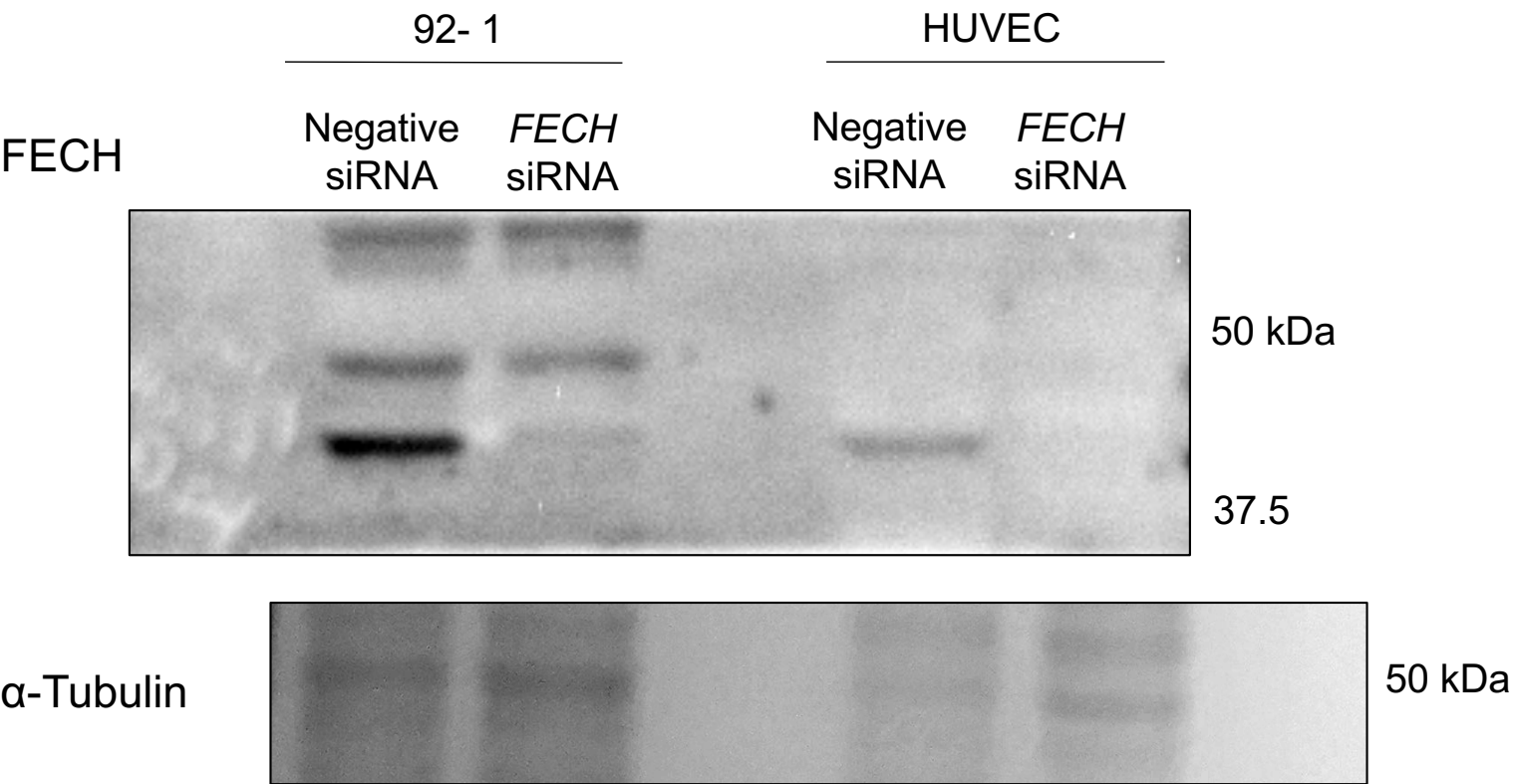

Supplement: Supplementary file 3 — Source Data for Expanded View [file EMMM-9-786-s006.zip › EMM201606561V4_Figure_EV4_source_data.pdf]

**Fig. 1C**

Ag stain

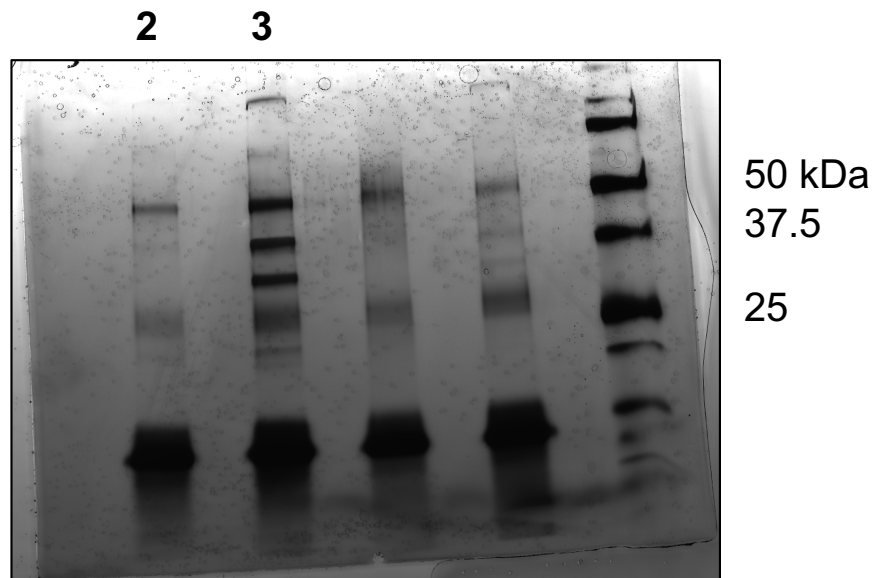

**Fig. 1D**

FECH

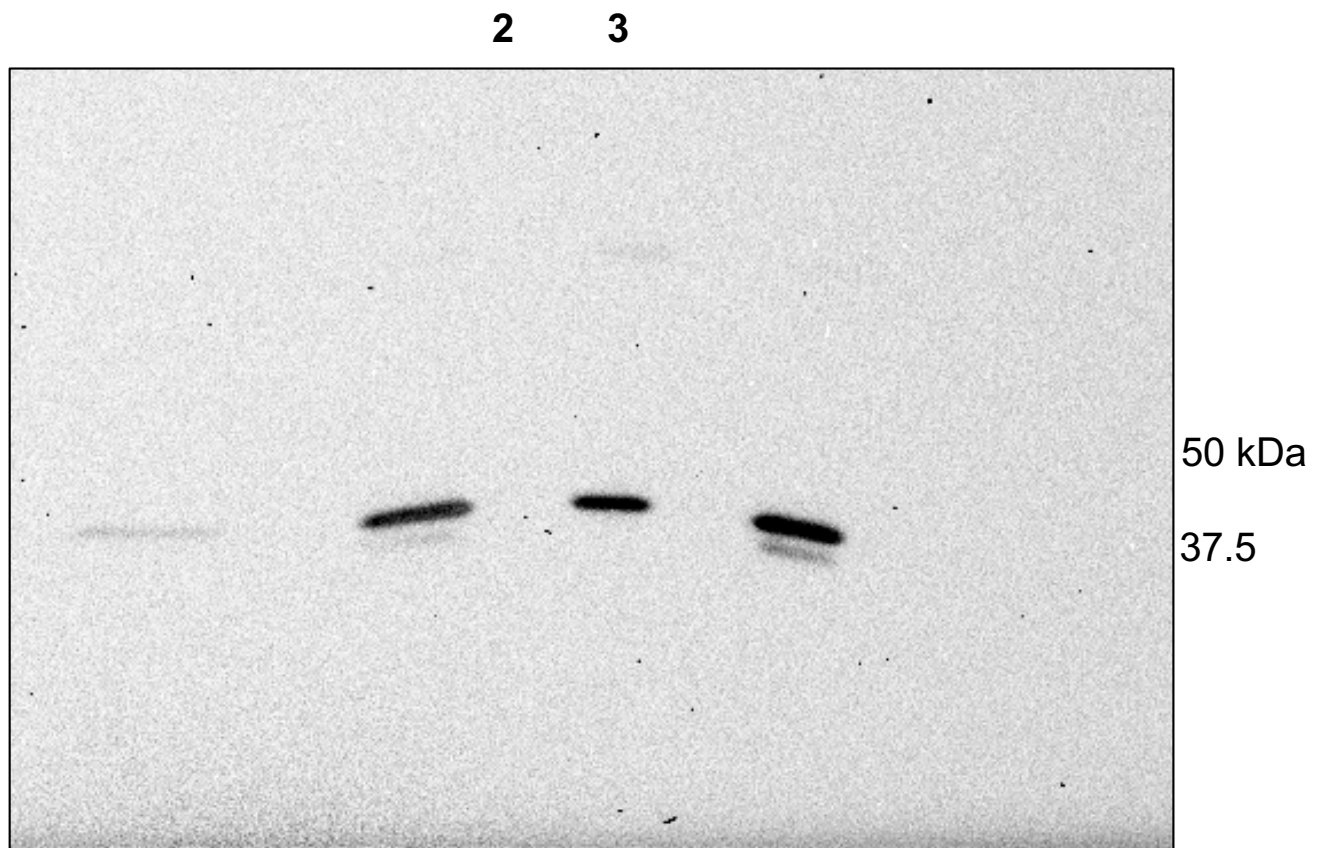

**Fig. 1E**

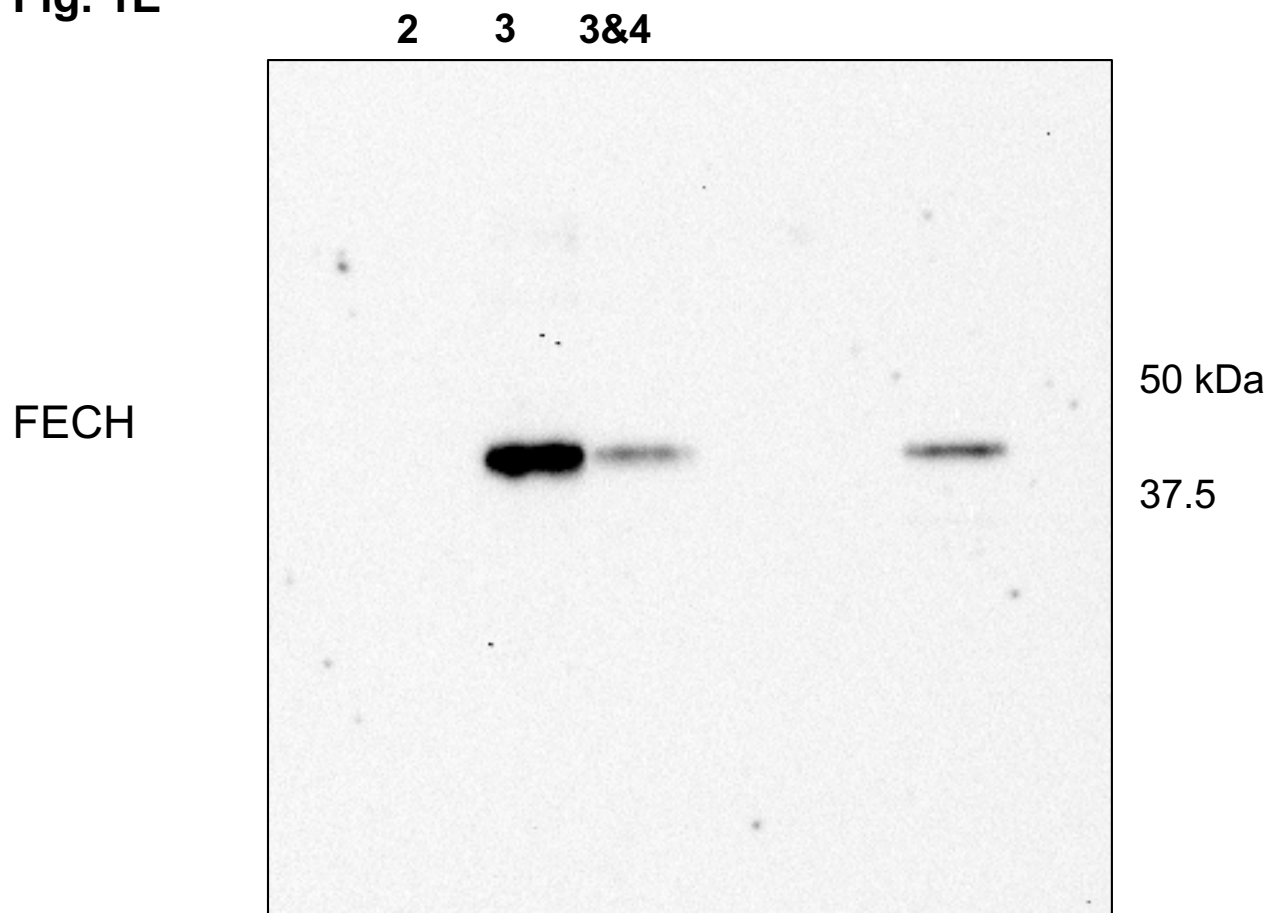

**Fig. 1F**

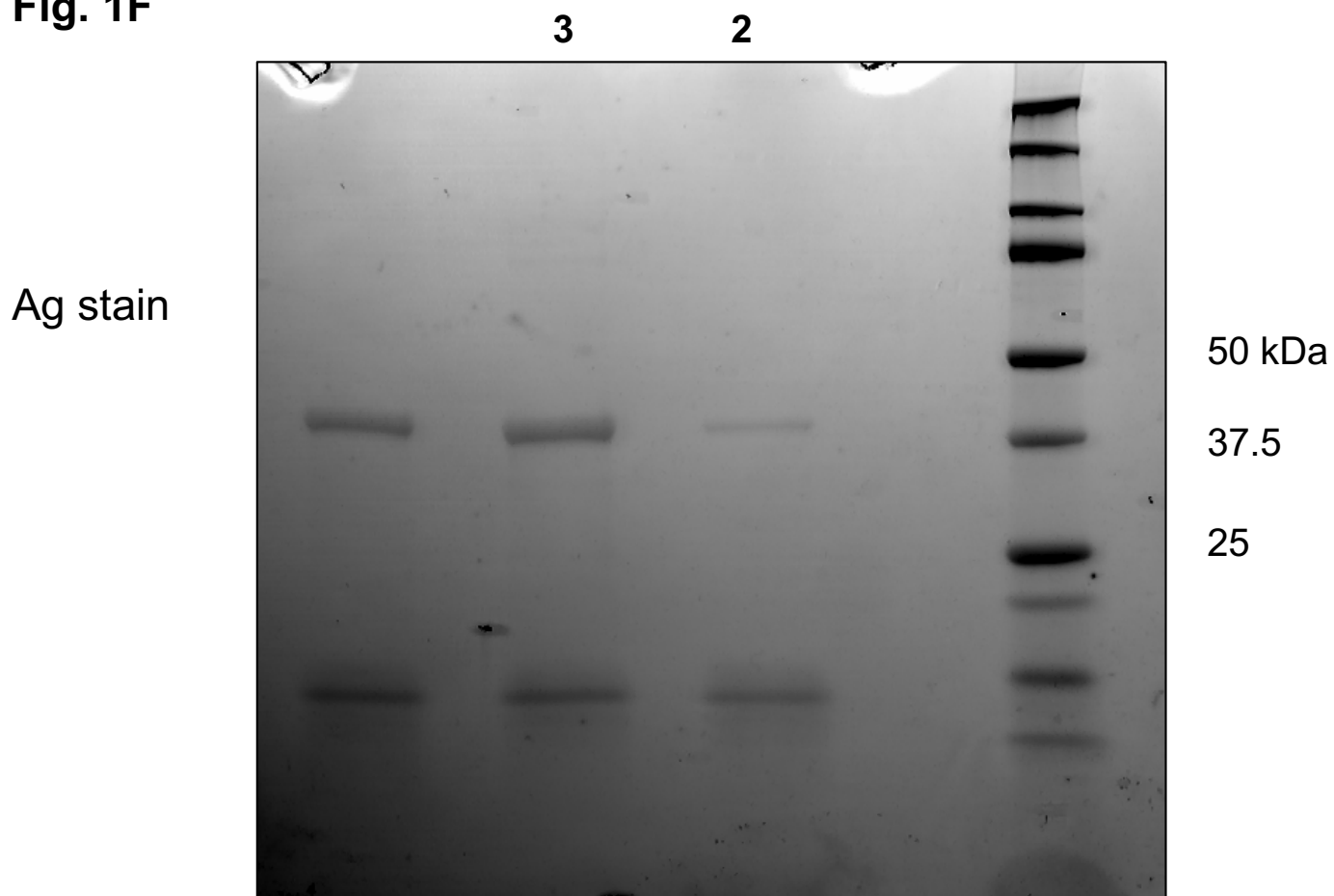

**Fig. 1G**

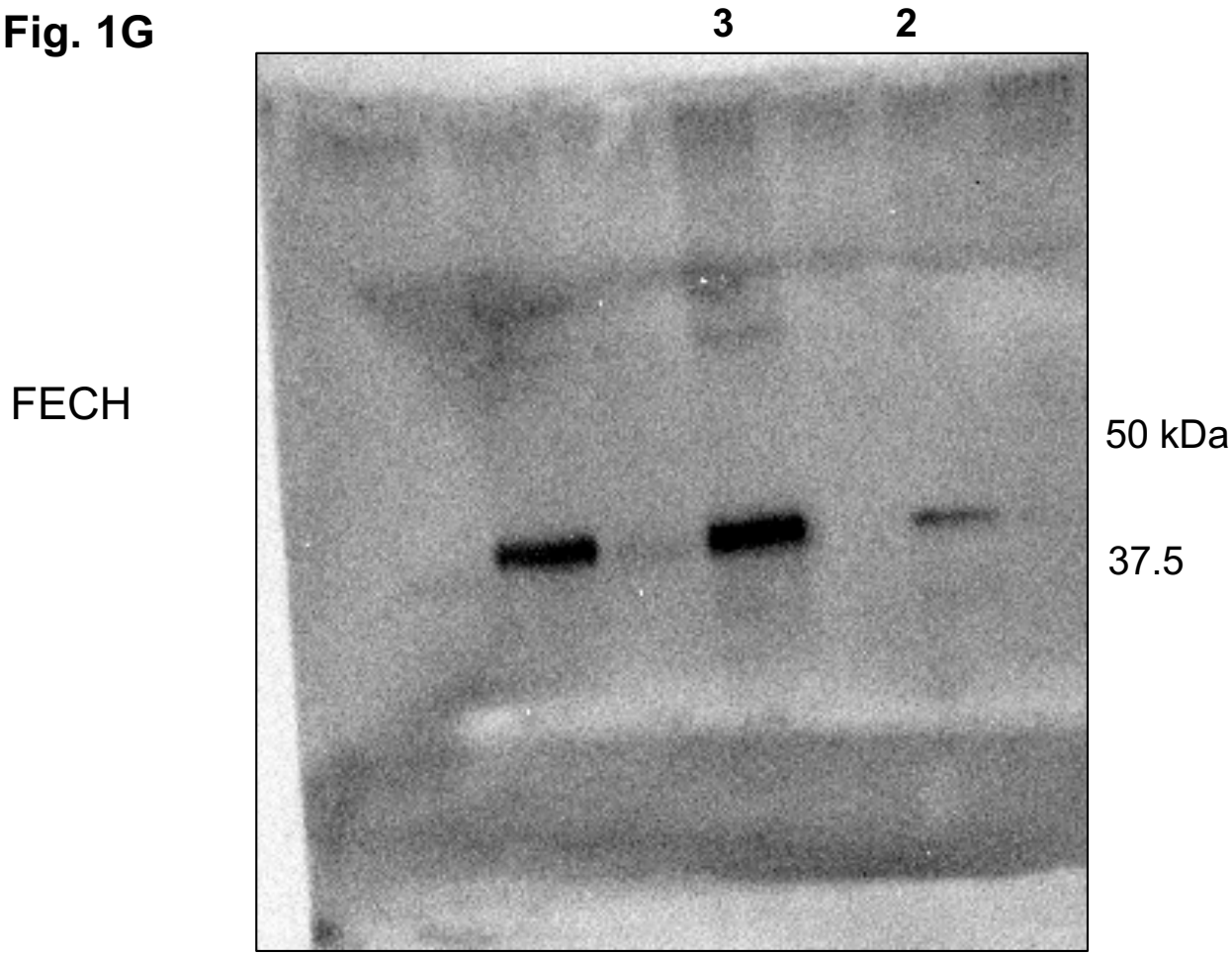

Supplement: Supplementary file 5 — Source Data for Figure 1 [file EMMM-9-786-s003.pdf]

Fig. 2A

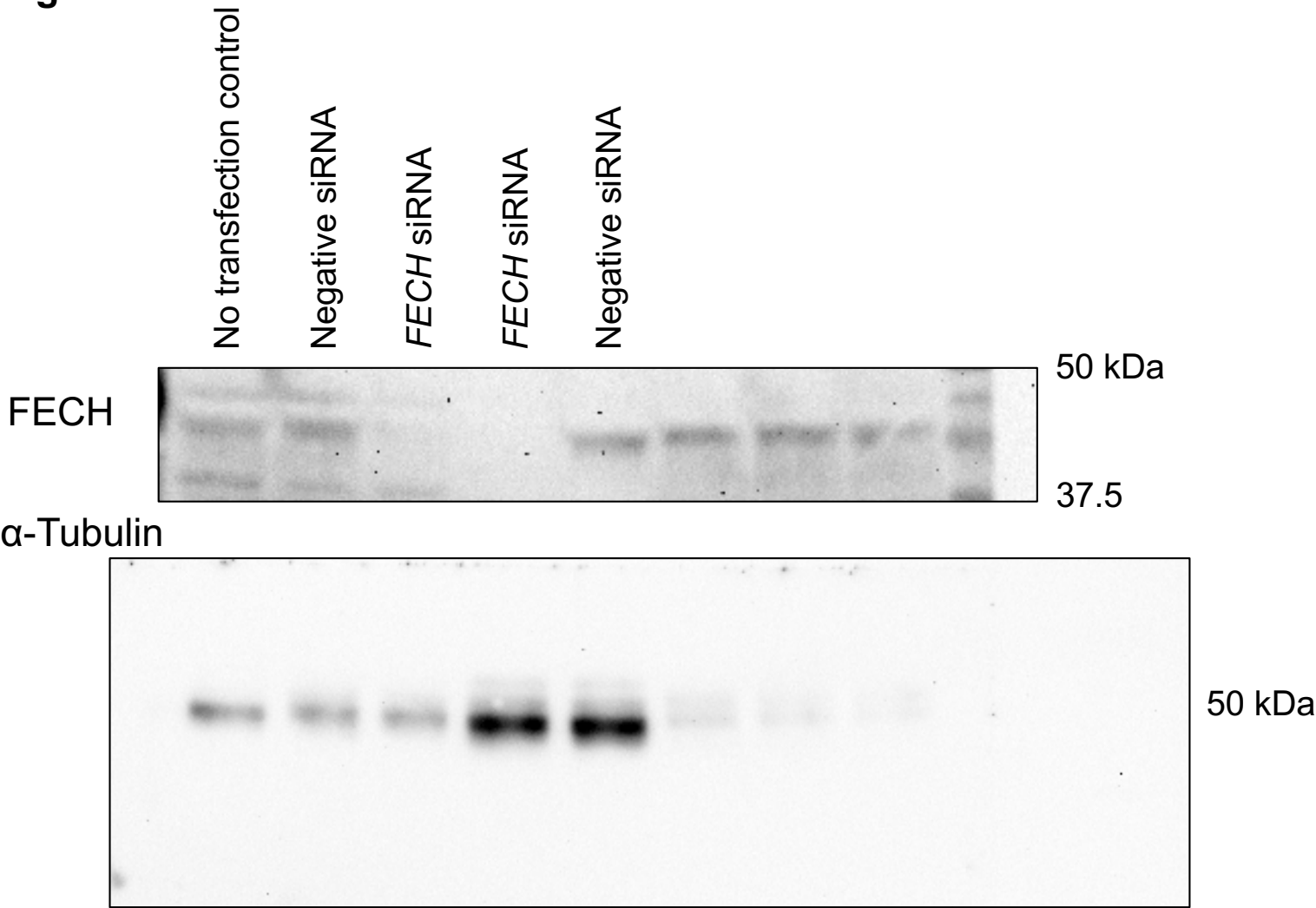

Supplement: Supplementary file 6 — Source Data for Figure 2 [file EMMM-9-786-s004.pdf]
