# Supplementary material for: Ferrochelatase is a therapeutic target for ocular neovascularization
Source: EMBO Mol Med. 2017 Apr 4;9(6):786–801. doi: 10.15252/emmm.201606561 (PMC5452042; doi:10.15252/emmm.201606561)
Supplement: Supplementary file 7 — Source Data for Figure 6 [file EMMM-9-786-s005.pdf]

Fig. 6A

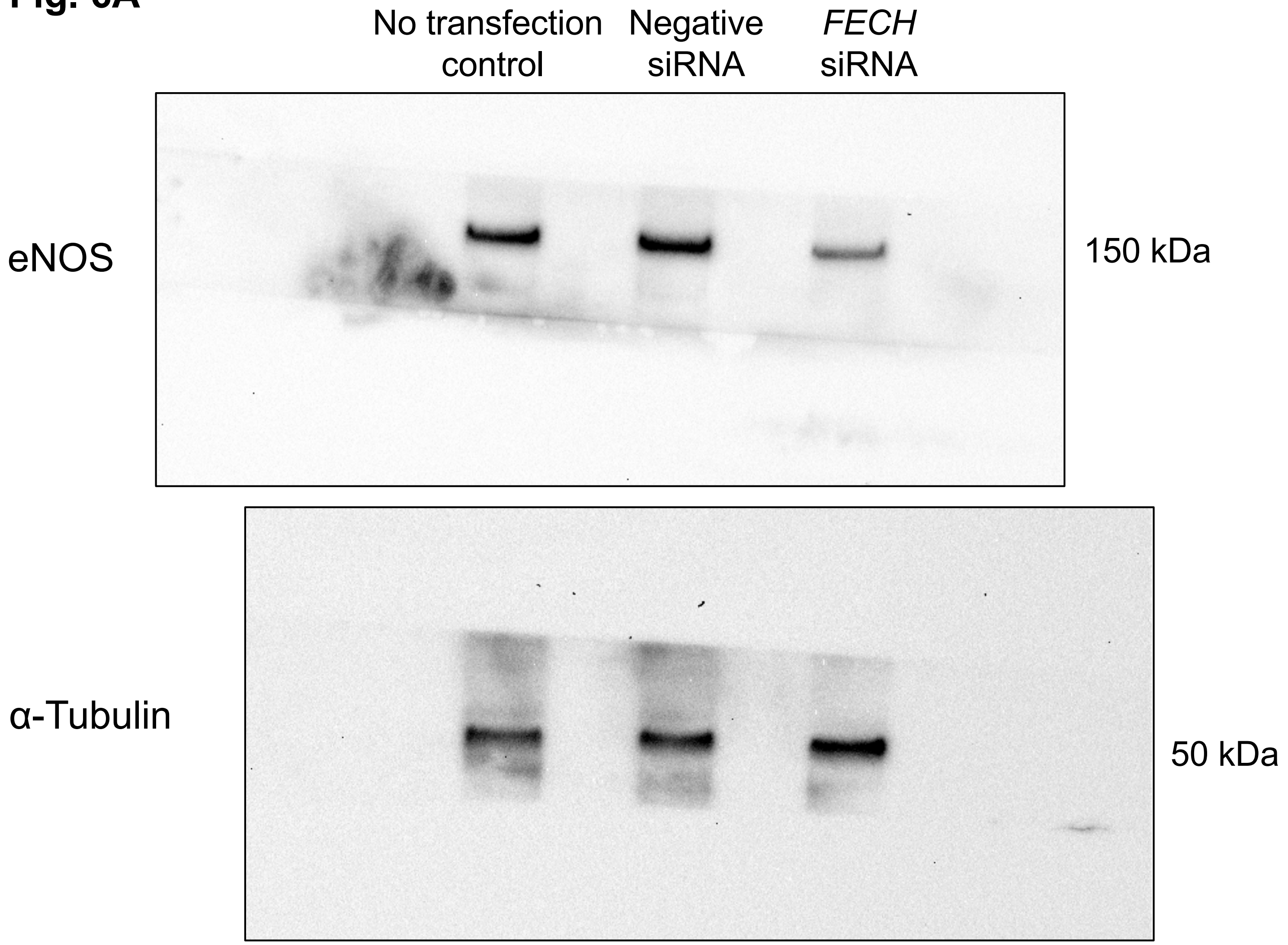

No transfection control
Negative siRNA
FECH siRNA
eNOS
150 kDa
α-Tubulin
50 kDa

Fig. 6C

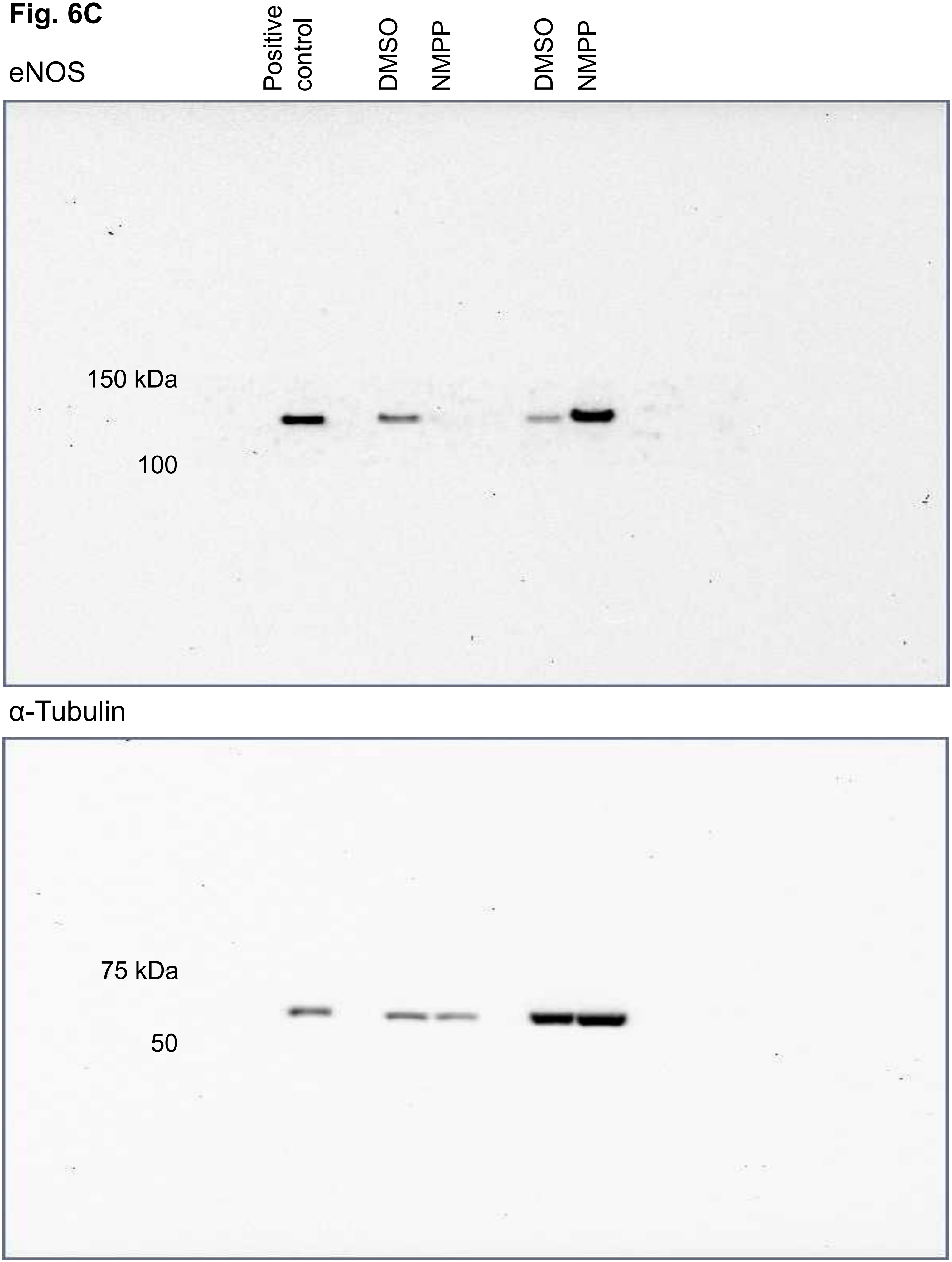
eNOS
Positive control
DMSO
NMPP
DMSO
NMPP
150 kDa
100
α-Tubulin
75 kDa
50

Fig. 6D

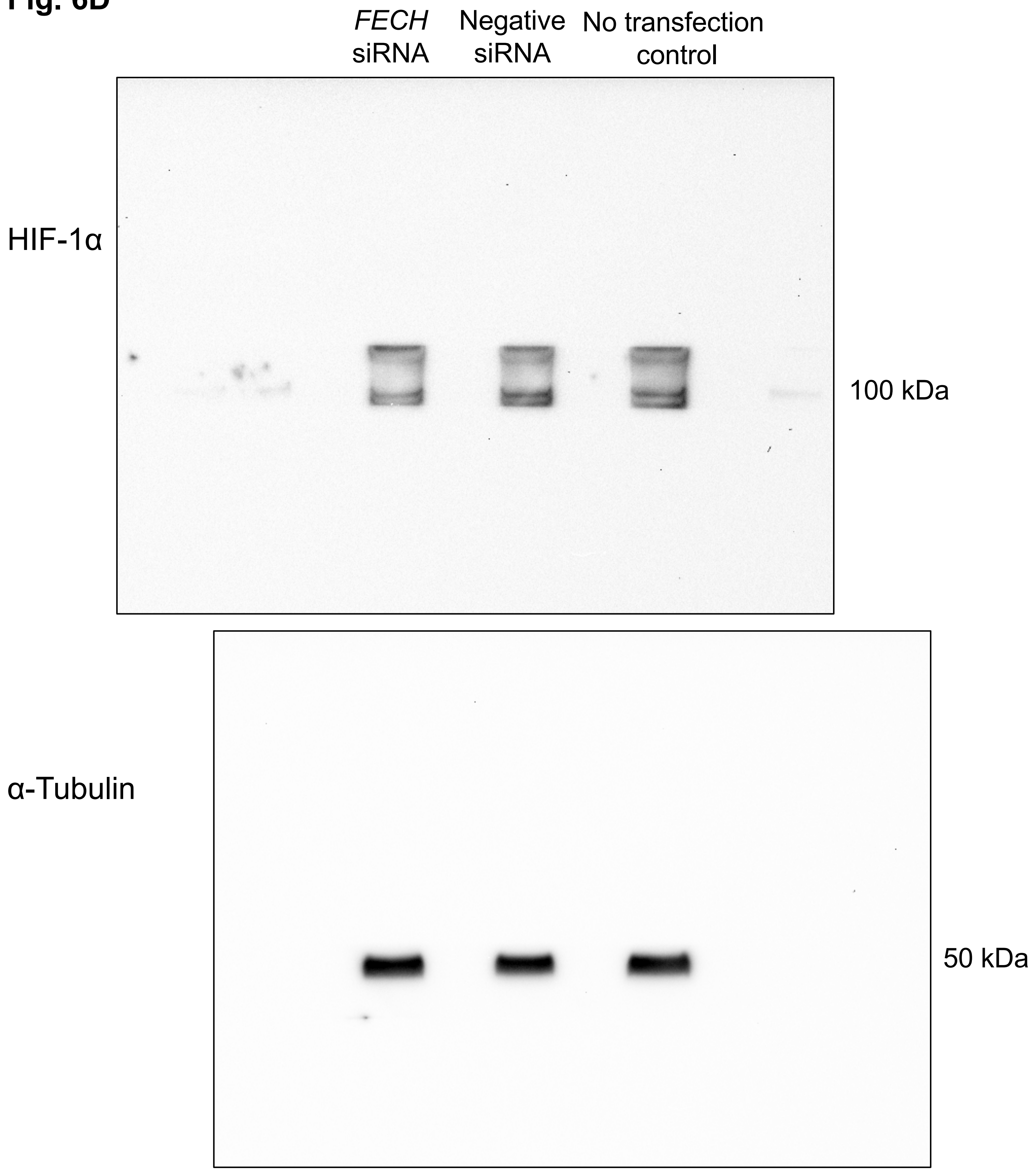

FECH siRNA
Negative siRNA
No transfection control
HIF-1α
100 kDa
α-Tubulin
50 kDa

Fig. 6E:
Top Panel

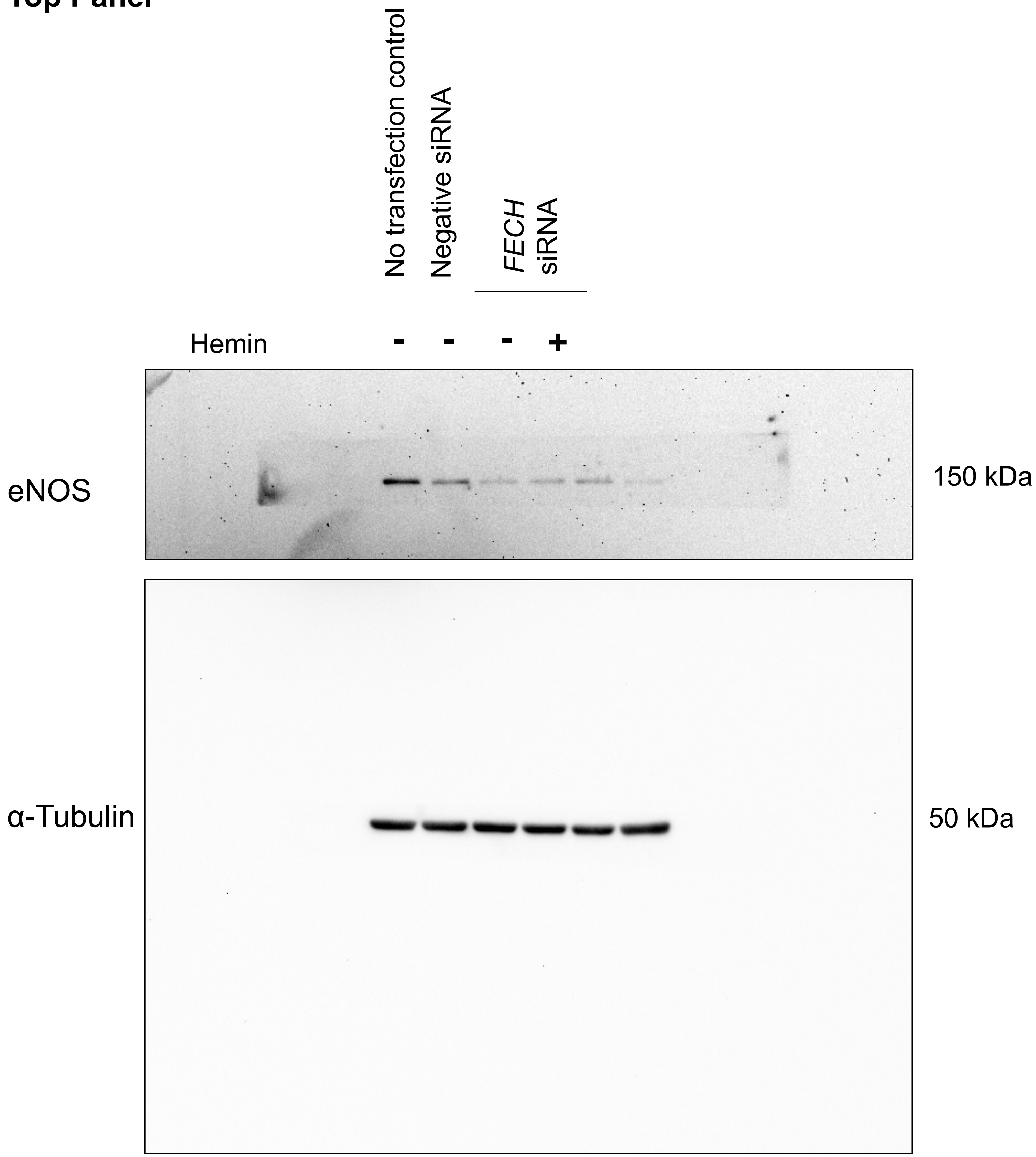

Fig. 6E:
Second Panel

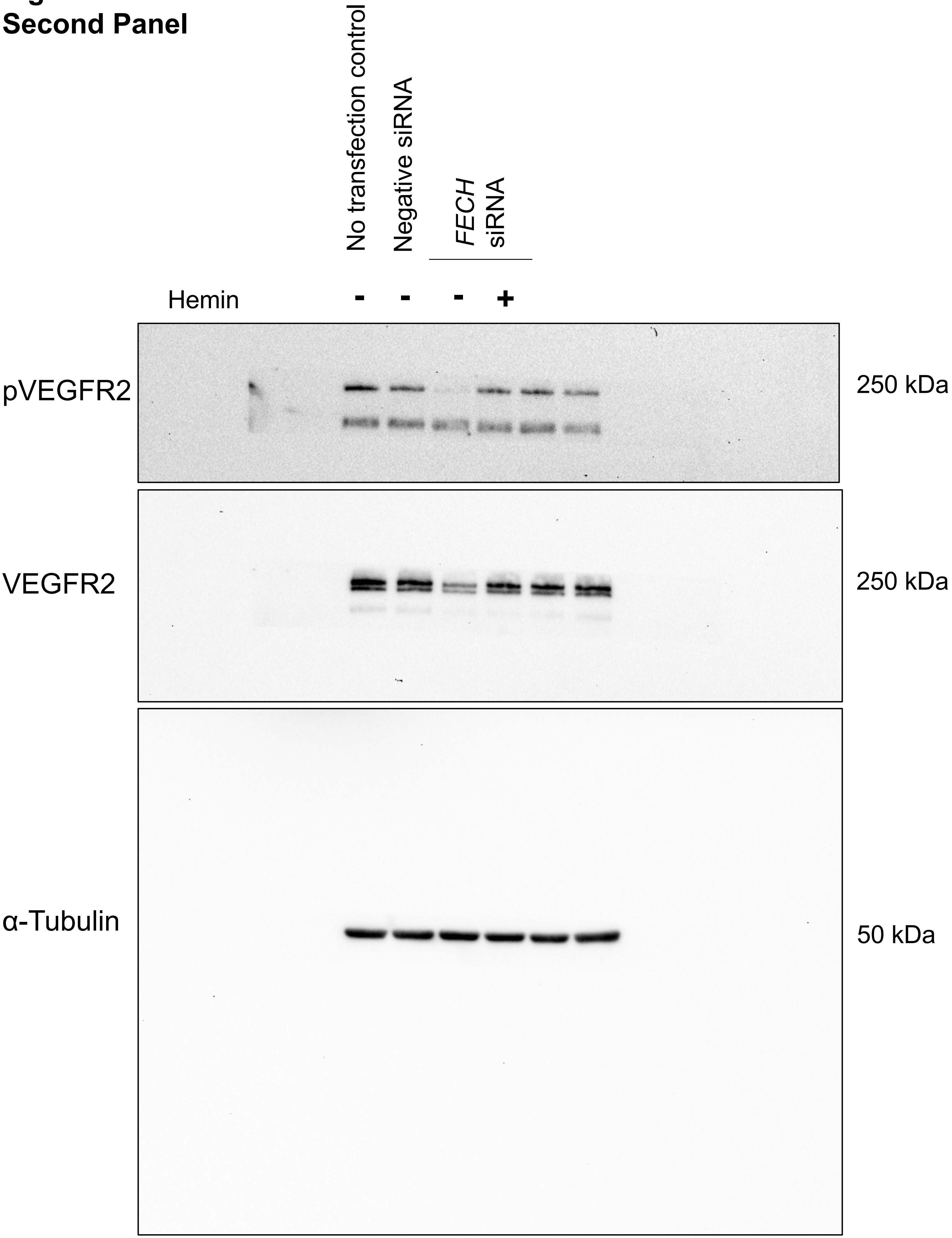

Fig. 6E:
Third Panel

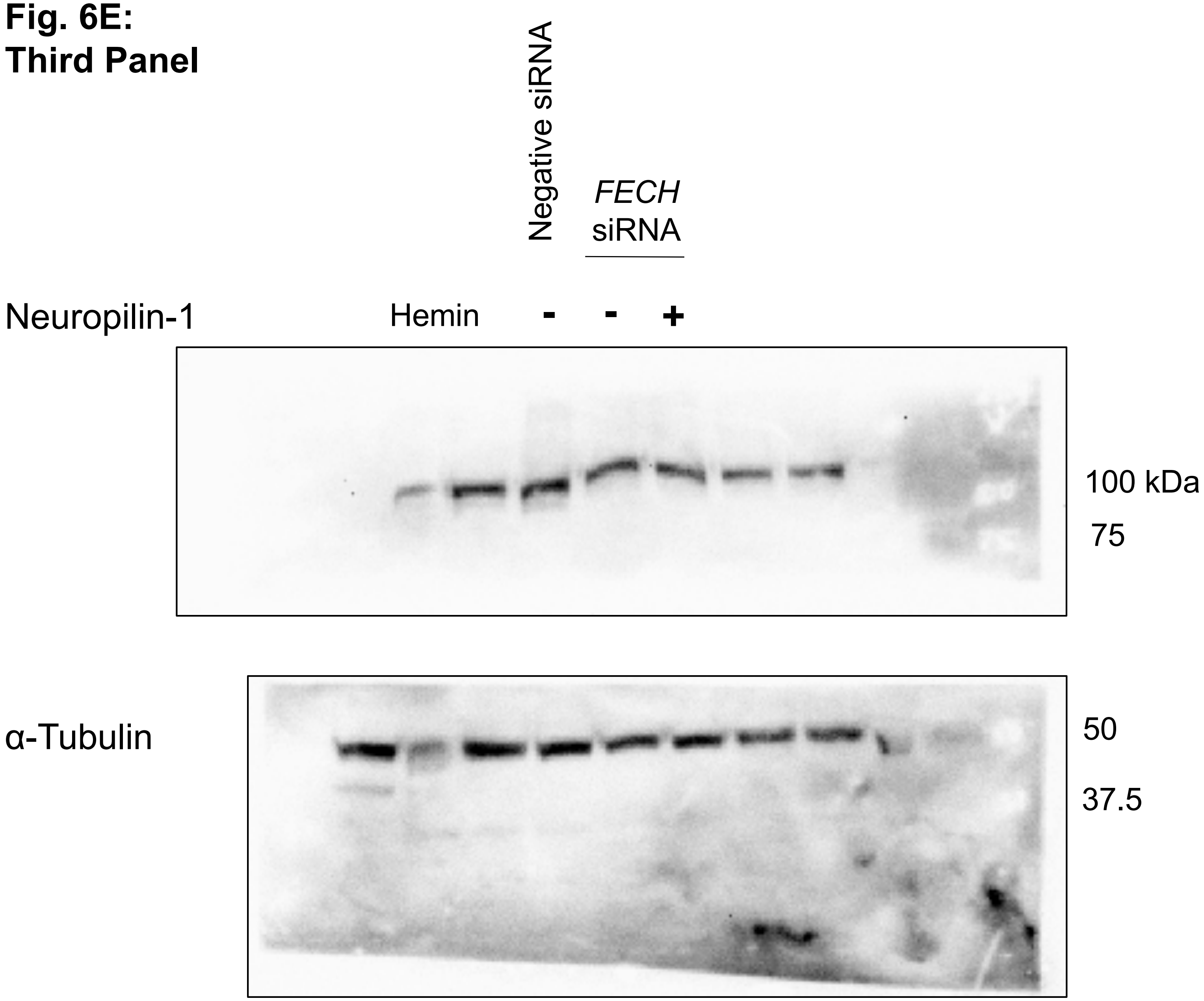

Fig. 6E:
Bottom Panel

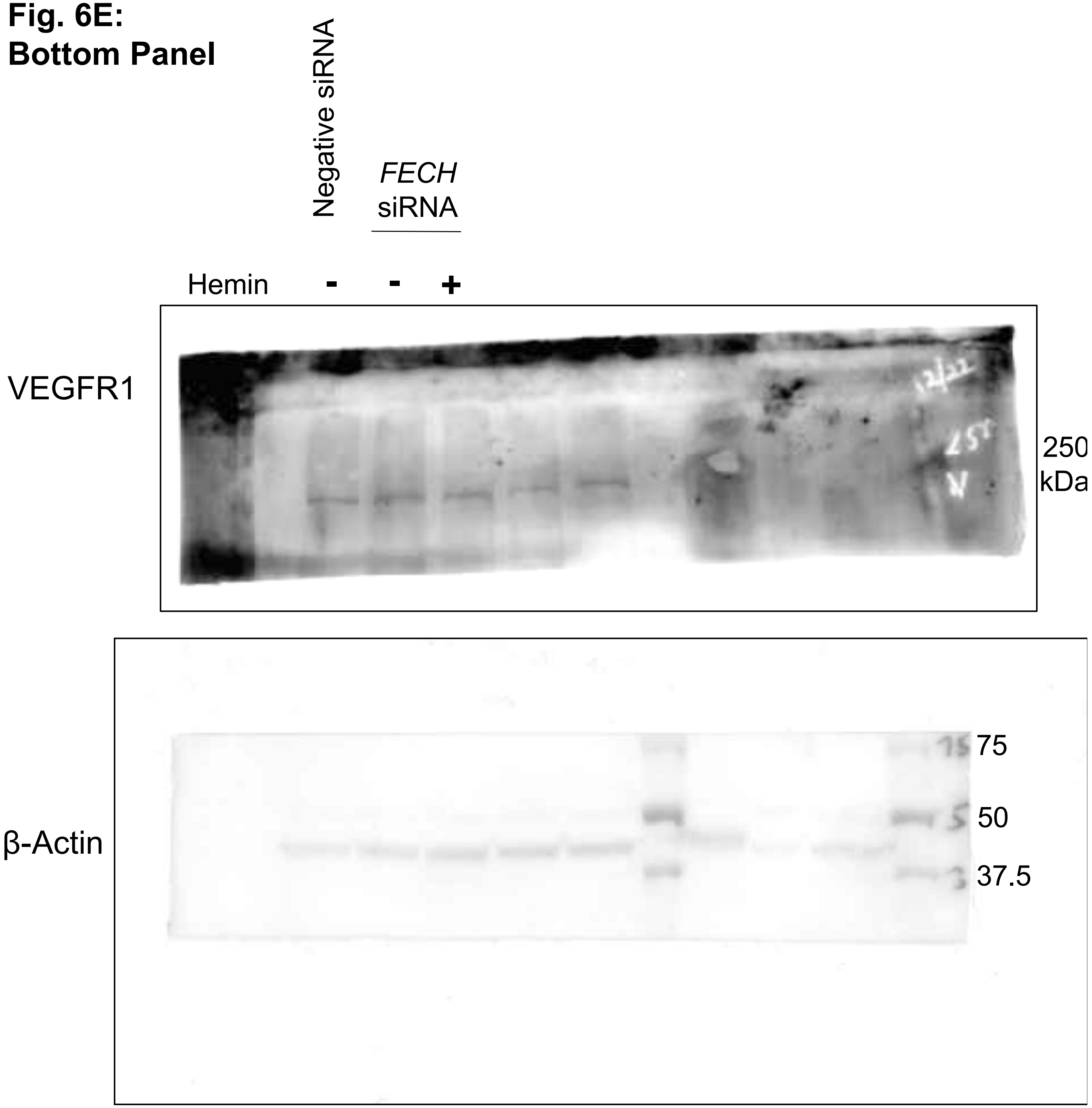

Fig. 6F

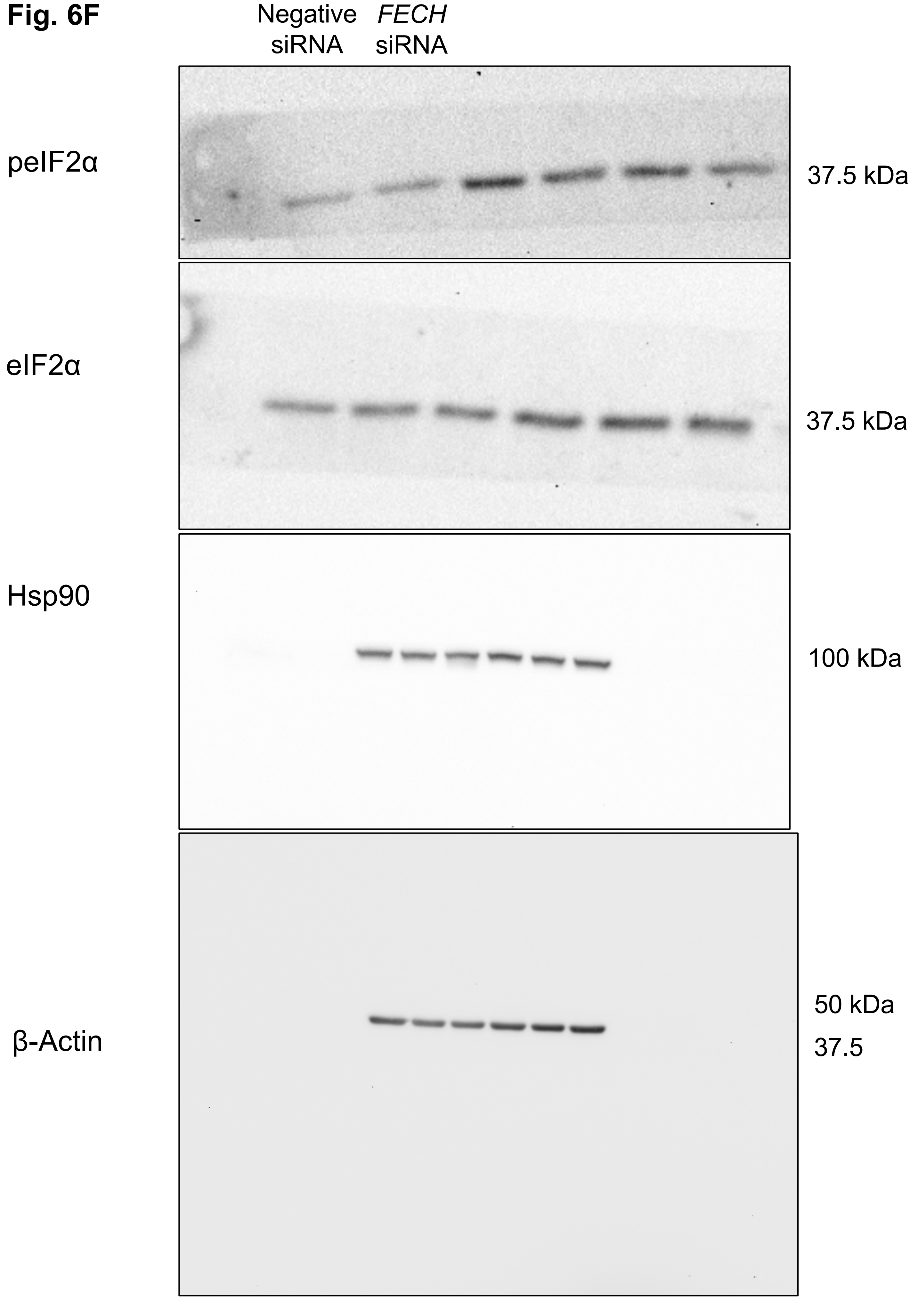
Negative siRNA
FECH siRNA
peIF2α
37.5 kDa
eIF2α
37.5 kDa
Hsp90
100 kDa
β-Actin
50 kDa
37.5
